# Structural and Photoluminescence Investigations of Tb^3+^/Eu^3+^ Co-Doped Silicate Sol-Gel Glass-Ceramics Containing CaF_2_ Nanocrystals

**DOI:** 10.3390/ma14040754

**Published:** 2021-02-05

**Authors:** Natalia Pawlik, Barbara Szpikowska-Sroka, Tomasz Goryczka, Joanna Pisarska, Wojciech A. Pisarski

**Affiliations:** 1 Institute of Chemistry, University of Silesia, 40-007 Katowice, Poland; barbara.szpikowska-sroka@us.edu.pl (B.S.-S.); joanna.pisarska@us.edu.pl (J.P.); wojciech.pisarski@us.edu.pl (W.A.P.); 2 Institute of Materials Engineering, University of Silesia, 41-500 Chorzów, Poland; tomasz.goryczka@us.edu.pl

**Keywords:** Tb^3+^/Eu^3+^ energy transfer, sol-gel processing, glass-ceramics, fluoride nanocrystals

## Abstract

In this work, the series of Tb^3+^/Eu^3+^ co-doped xerogels and derivative glass-ceramics containing CaF_2_ nanocrystals were prepared and characterized. The in situ formation of fluoride crystals was verified by an X-ray diffraction technique (XRD) and transmission electron microscopy (TEM). The studies of the Tb^3+^/Eu^3+^ energy transfer (ET) process were performed based on excitation and emission spectra along with luminescence decay analysis. According to emission spectra recorded under near-ultraviolet (NUV) excitation (351 nm, ^7^F_6_ → ^5^L_9_ transition of Tb^3+^), the mutual coexistence of the ^5^D_4_ → ^7^F_J_ (J = 6–3) (Tb^3+^) and the ^5^D_0_ → ^7^F_J_ (J = 0–4) (Eu^3+^) luminescence bands was clearly observed. The co-doping also resulted in gradual shortening of a lifetime from the ^5^D_4_ state of Tb^3+^ ions, and the ET efficiencies were varied from η_ET_ = 11.9% (Tb^3+^:Eu^3+^ = 1:0.5) to η_ET_ = 22.9% (Tb^3+^:Eu^3+^ = 1:2) for xerogels, and from η_ET_ = 25.7% (Tb^3+^:Eu^3+^ = 1:0.5) up to η_ET_ = 67.4% (Tb^3+^:Eu^3+^ = 1:2) for glass-ceramics. Performed decay analysis from the ^5^D_0_ (Eu^3+^) and the ^5^D_4_ (Tb^3+^) state revealed a correlation with the change in Tb^3+^–Eu^3+^ and Eu^3+^–Eu^3+^ interionic distances resulting from both the variable Tb^3+^:Eu^3+^ molar ratio and their partial segregation in CaF_2_ nanophase.

## 1. Introduction

In recent years, the materials doped with rare earths (RE^3+^) are considered to be indispensable in the development of optoelectronics, offering promising applications in LEDs [1], displays [2], lasers [3], or optical thermometry [4]. The proper adjustment of emission (i.e., generation of appropriate color purity and maintaining suitable luminescence lifetimes) usually requires the involvement of several RE^3+^ ions incorporated into the same host material [5,6,7,8,9,10,11,12]. Indeed, the mutual interactions between them—depending on the concentration of donor and acceptor as well as the type of host—allow for successful tailoring of the above-mentioned optical parameters.

Among numerous combinations of RE^3+^ in doubly- or triply-doped materials, the optical system consisting of Tb^3+^ and Eu^3+^ is a very promising strategy for the generation of multicolor luminescence, which plays a key role in the development of red-green-blue (RGB) optical materials. The Tb^3+^/Eu^3+^ energy transfer has been extensively explored and described in various types of phosphors, e.g., LaBWO_6_ [13], Tb_2_MoO_3_O_12_ [14], ScPO_4_ [15], or KAlP_2_O_7_ [16]; meanwhile, such studies have not been as common for glass-ceramic materials so far, where Tb^3+^ and Eu^3+^ ions could be distributed between the amorphous glassy host and crystal phase characterized by different decay rates. The evidence of Tb^3+^/Eu^3+^ ET is stated by the shortening of a lifetime from the ^5^D_4_ state of Tb^3+^ in the presence of acceptor ions (Eu^3+^). It is reported in the literature that the fluorescence decay becomes quicker with the increment of Eu^3+^ content, which accelerates the Tb^3+^/Eu^3+^ ET and makes it more efficient [17,18,19,20,21,22]. Furthermore, a comparative analysis of energy transfer efficiency, η_ET_, in precursor glasses and glass-ceramics is also carried out to demonstrate the impact of controlled crystallization on Tb^3+^/Eu^3+^ ET. Such studies were performed in excellent work by Chen et al. [19] for 44SiO_2_-28Al_2_O_3_-17NaF-(10-x)YF_3_-1TbF_3_-xEuF_3_ (x = 0, 0.1, 0.25, 0.5, 1) (mol%) glasses and derivative glass-ceramics fabricated at 670 °C. The η_ET_ for a glass containing 0.1 mol% Eu^3+^ achieved as low value as 1.39%, which finally grew to 30.28% for a glass containing 1 mol% Eu^3+^. Further, the authors have clearly proven that a crucial role in Tb^3+^/Eu^3+^ ET plays is glass crystallization, which results in significant growth in η_ET_ values from 16.63% (for glass-ceramic containing 0.1 mol% Eu^3+^) up to 47.70% (for glass-ceramic containing 1 mol% Eu^3+^). Similarly, an impact of controlled ceramization on Tb^3+^/Eu^3+^ ET behavior was also studied by Hu et al. [21], who found that η_ET_ increased from 8.7% for glasses with composition 45SiO_2_-20Al_2_O_3_-10CaO-24.04CaF_2_-0.05TbF_3_-0.01EuF_3_ (mol%) up to 14.0% for nano-glass-ceramic produced at 700 °C.

It should be pointed out that the majority of glass-ceramics containing Tb^3+^ and Eu^3+^ ions characterized and described in literature was prepared by the conventional melt-quenching method followed by controlled heat-treatment at the specified conditions of time and temperature [17,18,19,21,22,23,24,25,26]. An alternative route to the fabrication of glass-ceramics is the sol-gel technique, which offers quite easy fabrication of materials with complex compositions, which could be difficult to obtain via the melt-quenching technique [27,28,29,30,31]. Moreover, particular research attention has been focused on oxyfluoride glass-ceramics, which possess higher chemical and mechanical stability than fluoride glasses and lower phonon energies than oxide glasses. Among fluorides, the calcium fluoride, CaF_2_, is an optically isotropic crystal characterized by a broad region of transparency from 0.13 up to 9.5 μm, wide bandgap (12 eV), and relatively low phonon energy (~466 cm^−1^) [32,33,34]. Those features of CaF_2_ crystals are urgent to be a suitable medium for optically active rare earths, widely dedicated to fulfilling many sophisticated, active functions for optoelectronics. Indeed, the optical materials based on the CaF_2_ phase are frequently applied to generate an efficient up- [35] and down-conversion luminescence [36] or white light emission [37]. Therefore, such materials could be successfully predisposed for use in laser technologies [38], bio-imaging [39], or to increase the efficiency of solar cells [40]. Moreover, according to our previous results for sol-gel nano-glass-ceramics containing divalent metals fluorides, MF_2_ (M = Ca, Sr, Ba), singly-doped with Eu^3+^ ions, the most efficient segregation of Eu^3+^ inside fluoride crystal lattice, was reported for the SiO_2_-CaF_2_ system [41,42,43]. Indeed, a clear correlation was observed between the average decay time of the ^5^D_0_ state and growing difference in ionic radius of Eu^3+^ and each of individual M^2+^ cation in the following order: Ca^2+^ → Sr^2+^ → Ba^2+^ (SiO_2_-CaF_2_:Eu^3+^: τ_avg_ = 11.92; SiO_2_-SrF_2_:Eu^3+^: τ_avg_ = 7.77; SiO_2_-BaF_2_:Eu^3+^: τ_avg_ = 4.08 ms). Therefore, due to the efficient and long-lived luminescence in fabricated SiO_2_-CaF_2_:Eu^3+^ nano-glass-ceramics, it seems that this material could be considered as a very promising host to study the Tb^3+^/Eu^3+^ ET. Indeed, we reported interesting results for Tb^3+^/Eu^3+^ ET for sol-gel systems containing selected trivalent metals fluorides, MF_3_ (M = Y, La) [44]; hence, we performed such measurements for nano-glass-ceramics with divalent metal fluoride, CaF_2_. Moreover, to the best of our knowledge, the investigation of Tb^3+^/Eu^3+^ ET in oxyfluoride sol-gel glass-ceramics is rarely described in the available literature. Indeed, it was examined only in SiO_2_-SrF_2_ [20] and SiO_2_-BaGdF_5_ [45] sol-gel nano-glass-ceramics; however, there are no reports on the SiO_2_-CaF_2_ system so far. Due to the above reasons, it seems justified to study the Tb^3+^/Eu^3+^ ET in silicate sol-gel glass-ceramics containing CaF_2_ nanocrystals.

In this work, we fabricated and examined the series of sol-gel SiO_2_-CaF_2_ nano-glass-ceramics co-doped with Tb^3+^ and Eu^3+^ ions with the variable Tb^3+^:Eu^3+^ molar ratio (0.05:x, where x = 0.025, 0.05, 0.075, 0.1). The CaF_2_ phase was obtained via in situ crystallization from Ca(CF_3_COO)_2_ at as low a temperature as 350 °C, and its presence was verified using XRD measurements and TEM microscopy. The changes in photoluminescence behavior of fabricated sol-gel materials have been described in association with the variable Tb^3+^:Eu^3+^ molar ratio, as well as the structural transformation from amorphous xerogels into nano-glass-ceramics. Based on photoluminescence results, the interactions between Tb^3+^ and Eu^3+^ ions were systematically investigated. Indeed, a clear correlation was observed between the R/G ratio and energy transfer efficiency (η_ET_), as well as decay times of the ^5^D_4_ state (Tb^3+^) as the Tb^3+^:Eu^3+^ molar ratio gradually decreased. Additionally, the lifetimes of the ^5^D_0_ excited level of Eu^3+^ ions were also determined. The obtained sol-gel materials exhibited bright multicolor luminescence tuned when the Tb^3+^:Eu^3+^ molar ratio was changed.

## 2. Materials and Methods

The series of xerogels co-doped with Tb^3+^ and Eu^3+^ ions were synthesized using the sol-gel method described elsewhere [41,46]. All reagents were taken from Sigma Aldrich Chemical Company (St. Louis, MO, USA). After pre-hydrolysis of the mixture containing TEOS, ethyl alcohol, deionized water, and acetic acid in molar ratio equals 1:4:10:0.5 (90 wt.%), the solutions of Ca(CH_3_COO)_2_, Tb(CH_3_COO)_3_, and Eu(CH_3_COO)_3_ in water and trifluoroacetic acid (TFA) were added dropwise. For each sol-gel sample, a mixture of TFA and acetates was 10 wt.%, and the molar ratio equaled TFA:Ca(CH_3_COO)_2_:Tb(CH_3_COO)_3_: Eu(CH_3_COO)_3_ = 5:1:0.05:x (where x = 0, 0.025, 0.05, 0.075, and 0.1). The obtained sols were dried at 35 °C for seven weeks to form solid xerogels. The following xerogel samples were denoted as follows: XG_1Tb0.5Eu_ (x = 0.025), XG_1Tb1Eu_ (x = 0.05), XG_1Tb1.5Eu_ (x = 0.075), and XG_1Tb2Eu_ (x = 0.1). The glass-ceramic materials were obtained by controlled heat-treatment at 350 °C for 10 h. Such glass-ceramics were denoted as appropriate GC samples: GC_1Tb0.5Eu_, GC_1Tb1Eu_, GC_1Tb1.5Eu_, GC_1Tb2Eu_. The sol-gel samples singly-doped with Tb^3+^ ions were also prepared (XG_1Tb_, GC_1Tb_) to compare with the luminescence properties of Tb^3+^/Eu^3+^ co-doped materials.

The sol-gel network’s vibrational modes were identified using the Nicolet iS50 ATR spectrometer (Thermo Fisher Scientific, Waltham, MA, USA) in a frequency region 500–4000 cm^−1^. The X-ray diffraction analysis of fabricated xerogels and glass-ceramics was performed using an X’Pert Pro diffractometer supplied by PANalytical with CuK_α_ radiation (Almelo, The Netherlands). The microstructure of fabricated glass-ceramics was observed using the JEOL JEM 3010 electron transmission microscope operated at 300 kV (JEOL JEM 3010, Tokyo, Japan). The excitation and emission spectra, as well as luminescence decay curves were recorded on Horiba Jobin Yvon FluoroMax-4 spectrofluorimeter supplied with 150 W Xe lamp (Horiba Jobin Yvon, Longjumeau, France). The spectra were recorded with 0.1 nm resolution, and the decay curves were recorded with 2 μs accuracy. All structural and luminescence measurements were carried out at room temperature.

## 3. Results and Discussion

### 3.1. Structural Characterization: XRD, TEM, and IR Spectroscopy

In general, fabricated sol-gel materials’ structural properties strongly determine the local environment around Tb^3+^ and Eu^3+^, which are crucial in understanding any changes in their photoluminescence behavior (i.e., emission spectra and decay profiles). The detailed studies in this aspect (the structural evolution from sols, through gels, and xerogels, up to nano-glass-ceramics) were systematically investigated and described for the similar SiO_2_-LaF_3_:Eu^3+^ system in our previous work [47]. Therefore, to explain the luminescence features of fabricated SiO_2_-CaF_2_:Tb^3+^, Eu^3+^ samples, a brief comment on their structural properties was also presented below.

**Figure 1:**
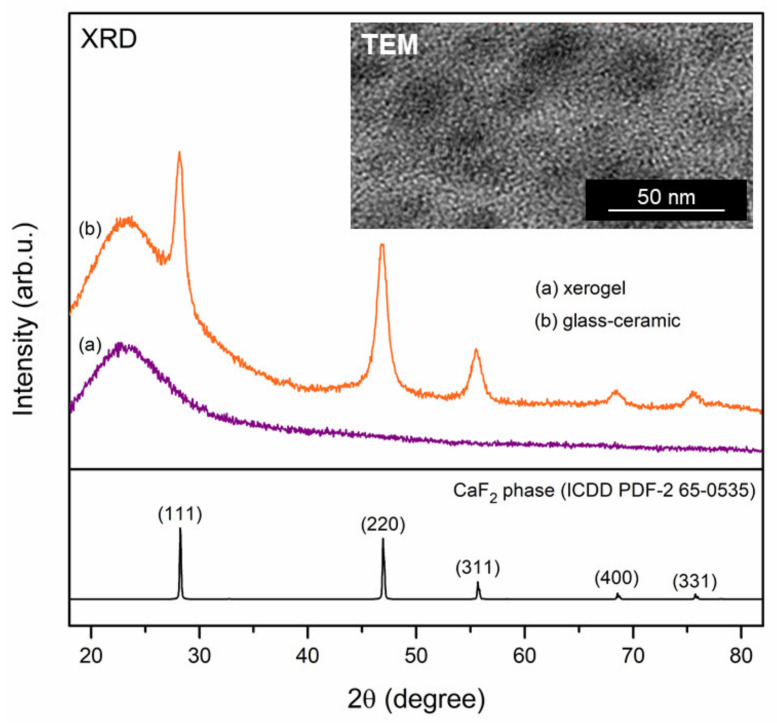
XRD patterns of XG_1Tb1Eu_ and GC_1Tb1Eu_ co-doped sol-gel materials. Inset shows the TEM image of glass-ceramic fabricated at 350 °C.

**Figure 2:**
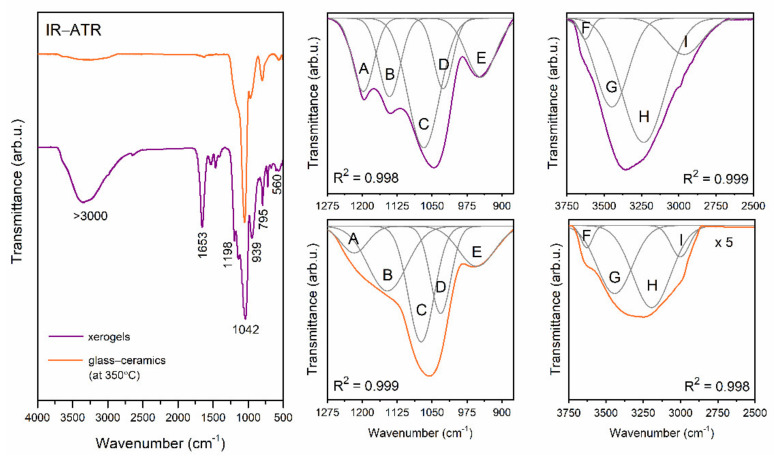
IR-ATR spectra recorded for xerogels and glass-ceramics. The deconvolution of bands characteristic for silicate host (1275–875 cm^−1^) as well as OH moieties and C–H bonds (3750–2500 cm^−1^) was also presented.

The performed heat-treatment of xerogels is responsible for both in situ crystallization of CaF_2_ nanophase (verified by XRD and TEM measurements, Figure 1) and evolution of the silicate sol-gel network (indicated by IR-ATR measurements, Figure 2). Such measurements were performed for representative XG_1Tb1Eu_ and GC_1Tb1Eu_ co-doped samples. As was demonstrated in Figure 1, a broad halo pattern was recorded for xerogel, which indicates an amorphous nature without long-range structural order. The diffraction reflexes characteristic for the CaF_2_ phase crystallized in Fm3m space group (ICDD, PDF-2 No. 65-0.0535) were observed after controlled ceramization. The broadening of recorded diffraction lines indicates the crystallization of the CaF_2_ phase in nanoscale, the average crystal size of which was estimated to be 8.1 ± 0.4 nm from the Scherrer formula:

(1) $$D\;=\;\frac{K\lambda }{B\cos\theta }$$

where D is the crystal size, K is a constant value (in our calculations, we took K = 1), λ is the X-ray wavelength (1.54056 Å, CuK_α_), B is a half of the diffraction line, and θ is the diffraction angle [48]. Another method that allowed us to determine the crystallites’ size, which is an extension of the Scherrer equation, was the Williamson–Hall method, based on plotting the following dependence for several reflexes derived from the same crystalline phase:

(2) $$\beta \cos\theta =\;\frac{K\lambda }{D}\;+\;4\left(\frac{\Delta a}{a}\right)\sin\theta$$

in which β is a half of the diffraction line and (Δa/a) refers to the lattice deformation [49]. From the Williamson–Hall plot’s linear fit, the crystallite size was estimated to be 16.6 ± 1.5 nm (the “chi-square” regression coefficient was equaled to 0.97). Since the Williamson–Hall approach considers crystal imperfections and lattice distortion, as well as apparatus factors, this method allowed us to determine the mean crystallite size more reliably than the Scherrer method (indeed, the latter does not consider such distortions of the crystal lattice). Indeed, the size of CaF_2_ nanocrystals from the TEM image (inset of Figure 1) was in more prominent agreement with the data obtained by the Williamson–Hall method (16.6 ± 1.5 nm) than by the Scherrer equation (8.1 ± 0.4 nm). It should be noted that the average crystal size estimated by the Williamson–Hall method was two-fold larger than from the Scherrer equation. Taking into account the factors related to the prepared sample itself, we suppose that one of the reasons for the discrepancy in the values estimated by various methods, apart from the differences in the ionic radii of Ca^2+^ (1.00 Å) [50], Tb^3+^ (1.04 Å), and Eu^3+^ (1.07 Å) [51], could be related to the charge compensation when trivalent cations were substituting divalent Ca^2+^ in the CaF_2_ crystal lattice. Indeed, to balance an excess of the positive charge introduced by the RE^3+^ ion, the F^-^ anions were distributed in interstitial positions [52]. Such interstitial F^-^ anions induced a local distortion in the crystal lattice due to the repulsion between them. Therefore, we assumed that the charge compensation effect might contribute to the identified difference in crystal size.

Besides the crystallization of the CaF_2_ nanophase, the track of structural changes inside the sol-gel host is also important to explain Tb^3+^ and Eu^3+^ dopant ions’ luminescence properties. The sol-gel systems were dynamic during controlled heat-treatment at 350 °C performed for the next 10 h because it induced evaporation of residual solvents (water and organic liquids) from a microporous structure and polycondensation reaction with the participation of Q^2^ ([Si(O_1/2_)_2_O_2_]^−2^), Q^3^ ([Si(O_1/2_)_3_O]^−^), and Q^4^ units ([Si(O_1/2_)_4_]) (in the description “O_1/2_” is corresponding to each oxygen atom, which is involved in the formation of Si–O–Si bridge; however, “O” is according to a non-bridging oxygen atom; therefore higher value of “n” index is, the less Si–OH unreacted groups are). To examine such structural evolution, the IR-ATR spectra were recorded in the frequency region from 500 to 4000 cm^−1^ (Figure 2), and the bands were assigned to appropriate vibrational modes based on literature data [53,54,55,56,57,58]. Generally, we distinguished three primary regions classified by the functional groups causing the characteristic vibrations: 3750–2500 cm^−1^ (OH groups and C–H bond), 1820–1510 cm^−1^ (C=O bond, Si–OH groups, and adsorbed H_2_O), and 1275–500 cm^−1^ (silicate host). For a more in-depth interpretation of oscillations that occurred in the sol-gel network, deconvolution was performed. The peak fitting during the deconvolution was done using a Gauss function with a squared regression coefficient of R^2^ ≥0.998.

Firstly, we analyzed the origin of deconvoluted bands (denoted as A–E) identified within the 1275–620 cm^−1^ spectral region. It was established that band A (~1200 cm^−1^) was corresponding to the TO_4_ mode of created Si–O–Si siloxane bridges, band B (~1140 cm^−1^) was correlated with oscillations within Q^4^ structural units ([Si(O_1/2_)_4_]), and band C (~1070 cm^−1^) could be associated with the TO_3_ mode of Si–O–Si siloxane bridges. Bands D (~1030 cm^−1^) and E (~960 cm^−1^) were related to Q^3^ and Q^2^ units’ oscillations, respectively [53,54,56]. According to the literature, bands A and B could also be interpreted as vibrations originated from the C–F bond inside –CF_3_ groups in trifluoroacetates [55]. It was clearly visible that the intensities of bands A, B, and E decreased during the transformation of xerogels into glass-ceramics. Indeed, since Ca(CF_3_COO)_2_ underwent thermal decomposition during controlled ceramization [41], the vibrations from the C–F bond disappeared for glass-ceramics, and bands A and B should have originated only from vibrations inside the Si–O–Si siloxane bridges. Further, a decrease in the intensity of band E clearly pointed to the conversion of Q^2^ structural units into Q^n^ (n = 3, 4) units as a consequence of polycondensation reaction. The additional weak bands located at the 616 and 561 cm^−1^ frequency region indicated some cyclic structures inside the sol-gel host [53,56].

**Figure 3:**
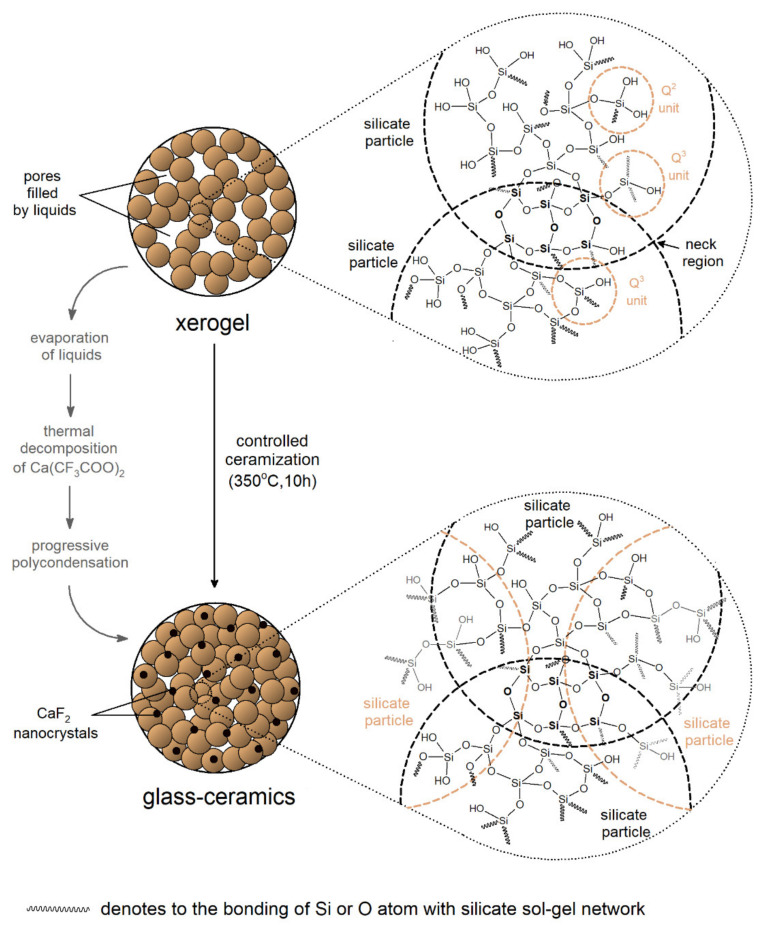
Graphical representation of structural transformation during controlled ceramization at 350 °C.

Finally, we analyzed the broad band recorded in a frequency region from 3750 up to 2500 cm^−1^. The deconvolution revealed the presence of three bands originated from different types of OH moieties: Geminal or vicinal Si–OH groups (~3630 cm^−1^, band F), hydrogen-bonded Si–OH groups (~3450 cm^−1^, band G), and hydrogen-bonded OH groups originated from residual water and organic compounds (~3200 cm^−1^, band H). It should be noted that deconvolution also revealed the fourth component band (~2990 cm^−1^, band I), which corresponded to the vibrations of C–H bonds [53,54,56]. It was observed that an indicated broad band was much more intense for xerogels than for glass-ceramics (indeed, to show the deconvolution better, the band’s intensity was fivefold enlarged). In fact, such a relatively strong band for xerogels was a consequence of “trapping” of water and organic liquids inside pores via hydrogen-bonding created with unreacted Si–OH groups. During ceramization, the band was significantly reduced, which clearly evidenced successful evaporation of water and organic liquids as well as a continuation of polycondensation reaction. The conclusions from the above observations could also be confirmed by the behavior of the infrared signal located at ~1660 cm^−1^, which was attributed to the vibrations of the C=O bond, Si–OH surface groups, and adsorbed water [55,57,58]. The indicated band was well-observable for xerogels, and it almost completely disappeared for glass-ceramics. According to IR and XRD results, the graphical visualization of sol-gel evolution during performed ceramization at 350 °C, as presented in Figure 3.

### 3.2. Luminescence Properties of Fabricated Sol-Gel Materials

#### 3.2.1. Determination of Local Symmetry Using Spectroscopy of Eu^3+^ Ions as Spectral Probes

**Figure 4:**
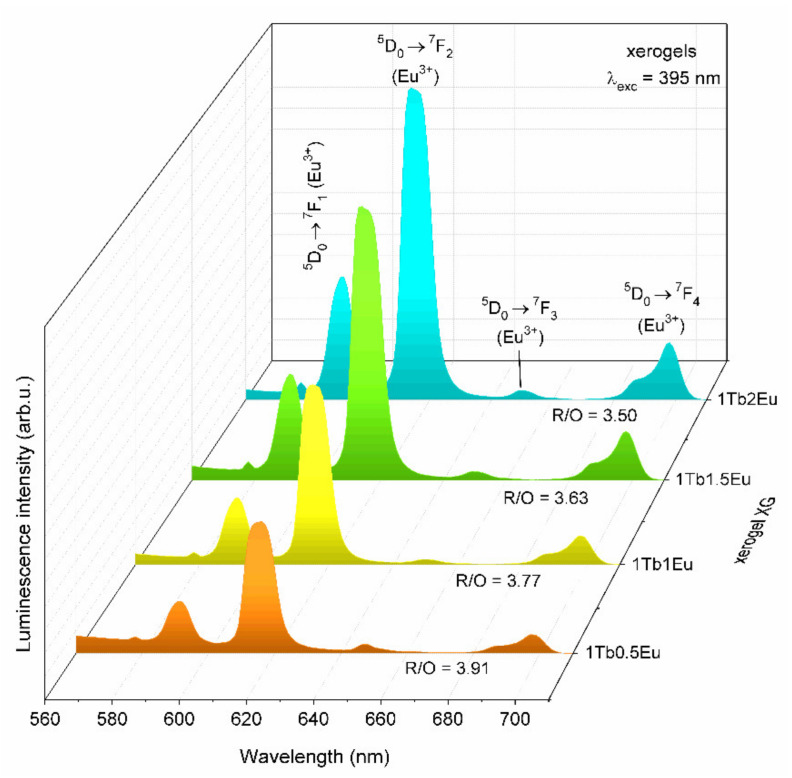
The emission spectra of XG_1TbxEu_ xerogels co-doped with Tb^3+^/Eu^3+^ ions recorded at λ_exc_ = 395 nm.

The emission spectra recorded for fabricated XG_1TbxEu_ xerogels using λ_exc_ = 395 nm excitation line were shown in Figure 4. The characteristic ^5^D_0_ → ^7^F_J_ luminescence bands of Eu^3+^ ions were detected in the reddish-orange light area and their maxima were located at following wavelengths: 578 (J = 0), 592 (J = 1), 615 (J = 2), 647 (J = 3), and 698 nm (J = 4). A gradual increase in their intensity was observed, when the Tb^3+^:Eu^3+^ molar ratio systematically changed from 1:0.5 to 1:2. It is clearly visible that among series of recorded bands, the ^5^D_0_ → ^7^F_2_ one was the most prominent line for all xerogels before their controlled ceramization, despite the Tb^3+^:Eu^3+^ molar ratio. Indeed, Eu^3+^ ions were frequently used as spectral probes due to the characteristic nature of their transitions. The ^5^D_0_ → ^7^F_1_ is a magnetic-dipole transition (MD) in nature, the intensity of which is rather independent of the host. In contrast, the ^5^D_0_ → ^7^F_0.2–4_ are electric-dipole transitions (ED) known to be forbidden by the Laporte selection rule and may occur due to mixing the 4f orbitals with the opposite parity at the low-symmetry sites. Among ED transitions, the ^5^D_0_ → ^7^F_2_ one has a hypersensitive nature, and its intensity is easily affected by the local vicinity: It is promoted in low-symmetric frameworks; meanwhile, it is inhibited in more symmetric environments. Hence, we could infer about the symmetry based on the ratio of integrated intensities of the bands mentioned above, which is well-known in literature as the R/O ratio (I(^5^D_0_ → ^7^F_2_)/I(^5^D_0_ → ^7^F_1_)) [59,60]. XRD results confirmed the amorphous nature of xerogels without long-range order, so we expected relatively high asymmetry in the immediate vicinity of Eu^3+^. Indeed, the calculated R/O ratio values hesitated from 3.50 (XG_1Tb2Eu_) to 3.91 (XG_1Tb0.5Eu_). On this occasion, it should also be noted that comparability in calculated R/O ratio values clearly pointed to a chemically similar environment of Eu^3+^ ions in all synthesized xerogels.

**Figure 5:**
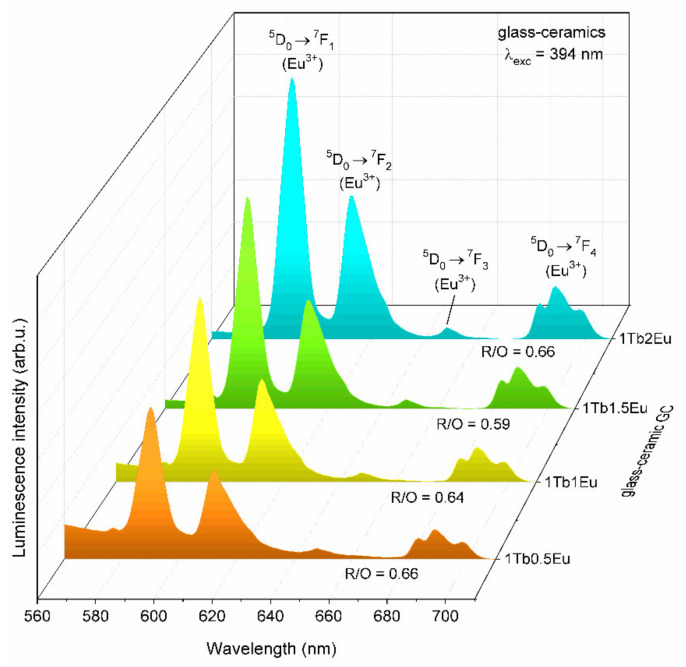
The emission spectra of GC_1TbxEu_ glass-ceramics co-doped with Tb^3+^/Eu^3+^ ions recorded at λ_exc_ = 394 nm.

The emission spectra recorded for GC_1TbxEu_ glass-ceramics upon excitation at λ_exc_ = 394 nm were presented in Figure 5. Similar as for xerogels, a series of characteristic bands corresponding to the intraconfigurational transitions within 4f^6^ manifold were registered: ^5^D_0_ → ^7^F_0_ (577), ^5^D_0_ → ^7^F_1_ (592), ^5^D_0_ → ^7^F_2_ (612), ^5^D_0_ → ^7^F_3_ (648) and ^5^D_0_ → ^7^F_4_ (683/689/698 nm). It was clearly observed that the intensity of the emission bands successfully increased with the growing content of Eu^3+^ ions (as the Tb^3+^:Eu^3+^ molar ratio changed from 1:0.5 to 1:2). For each glass-ceramic, the orange emission band corresponding to the ^5^D_0_ → ^7^F_1_ MD transition maintained the greatest intensity and dominated the ^5^D_0_ → ^7^F_2_ ED red band. Generally, an almost six-fold decline of R/O ratio value was observed, which equaled close to 0.64 after controlled ceramization (compared with xerogels for which the R/O ratio value was approximately equal to 3.70). Hence, such a decrease in the R/O ratio value pointed to significant changes in the symmetry in the immediate vicinity of Eu^3+^ ions, as well as a change in the nature of the bonding character from covalent to more ionic [61]. When the nearest framework of Eu^3+^ ions was more symmetric (which usually accompanied the migration of Eu^3+^ from an asymmetric amorphous structure without long-range order to crystalline lattice), the probability of the ^5^D_0_ → ^7^F_0,2–4_ electric-dipole transitions successfully decreased [60]. Indeed, the identified decrease in the R/O ratio value was strictly accompanied by partially entering of optically active ions into precipitated CaF_2_ fluoride crystal fraction with long-range order. In other words, such a decline was direct evidence that Ca^2+^ cations from fluoride crystal lattice were successfully substituted by Eu^3+^ ions. It should also be noted that a Stark splitting characteristic in the crystal-field was not observed because part of the Eu^3+^ ions was still distributed in an amorphous sol-gel host [60].

#### 3.2.2. Studies of Tb^3+^/Eu^3+^ Energy Transfer in Sol-Gel Materials with the Variable Tb^3+^:Eu^3+^ Molar Ratio

**Figure 6:**
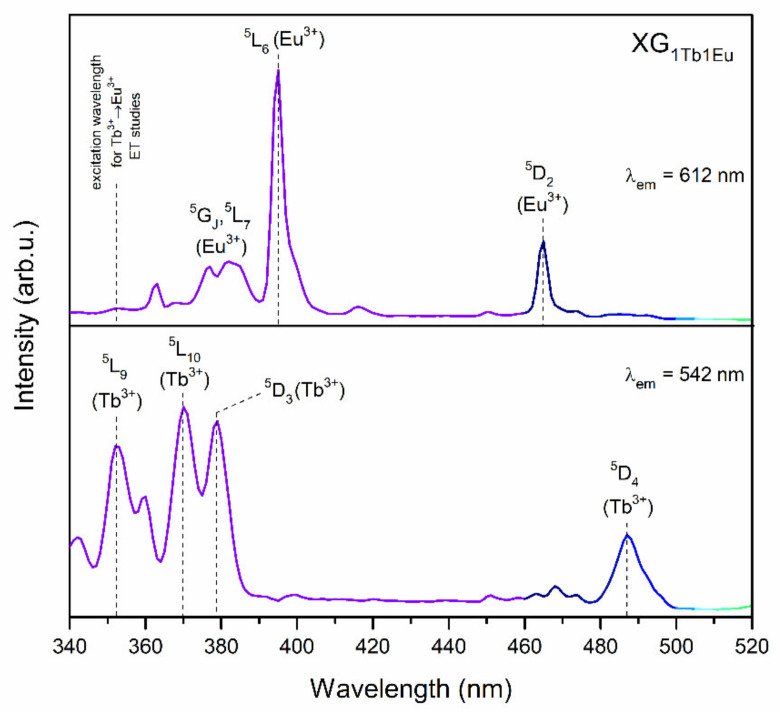
Excitation spectra recorded for red (λ_em_ = 612) and green (λ_em_ = 542 nm) emission lines for XG_1Tb1Eu_ xerogel.

To select the excitation line appropriate for Tb^3+^/Eu^3+^ ET studies, the photoluminescence excitation spectra for XG_1Tb1Eu_ co-doped representative xerogel were presented in Figure 6. The spectra were recorded for λ_em_ = 543 and λ_em_ = 612 nm emissions of Tb^3+^ (the ^5^D_4_ → ^7^F_5_ green line), and Eu^3+^ (the ^5^D_0_ → ^7^F_2_ red line), respectively. The recorded excitation bands of Tb^3+^ ions were associated to the following f-f intraconfigurational transitions: ^7^F_6_ → ^5^L_9_ (352), ^7^F_6_ → ^5^L_10_ (370), ^7^F_6_ → ^5^D_3_ (379), as well as ^7^F_6_ → ^5^D_4_ (487 nm). Simultaneously, the recorded bands were ascribed to the transitions of Eu^3+^ ions from the ^7^F_0_ ground state into the subsequent higher energy levels: ^5^D_4_ (363), ^5^G_J_, ^5^L_7_ (from 371 to 390), ^5^L_6_ (395), and ^5^D_2_ (465 nm). Since the ^7^F_6_ → ^5^L_9_ transition of Tb^3+^ ions did not coincide with any excitation peak of Eu^3+^, we decided to select the λ_exc_ = 351 nm excitation line to study the Tb^3+^/Eu^3+^ ET process.

**Figure 7:**
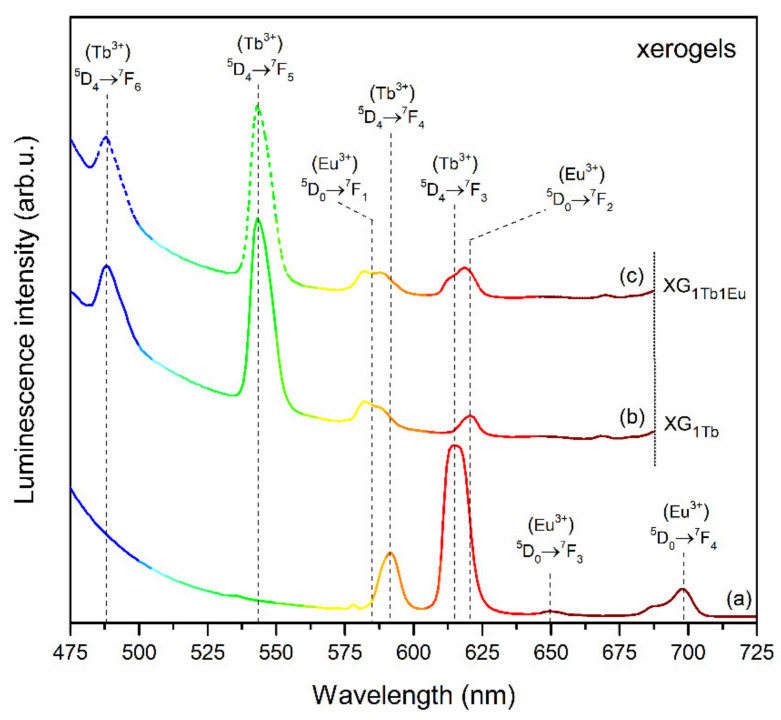
Emission spectra of: (**a**) XG_1Tb1Eu_ (λ_exc_ = 395), (**b**) XG_1Tb_ (λ_exc_ = 351), and (**c**) XG_1Tb1Eu_ (λ_exc_ = 351 nm).

The photoluminescence spectra recorded for XG_1Tb_ xerogel (upon excitation at λ_exc_ = 351 nm wavelength) as well as for the XG_1Tb1Eu_ co-doped representative sample (recorded under excitation at λ_exc_ = 395 and λ_exc_ = 351 nm lines) are depicted in Figure 7. The spectra recorded for the XG_1Tb_ sample revealed two emission bands in bluish-green spectral scope, i.e., ^5^D_4_ → ^7^F_6_ (488) and ^5^D_4_ → ^7^F_5_ (542 nm) of the predominant intensity, as well as two other emission bands of Tb^3+^ ions in the yellowish-red area: ^5^D_4_ → ^7^F_4_ (582) and ^5^D_4_ → ^7^F_3_ (620 nm). When the XG_1Tb1Eu_ co-doped sample was excited by the λ_exc_ = 395 nm line, only the characteristic 4f^6^-4f^6^ emission bands originated from Eu^3+^ ions (^5^D_0_ → ^7^F_J_, J = 0–4) were recorded. A tune in the excitation wavelength to λ_exc_ = 351 nm also led to the generation of the characteristic emission lines of Tb^3+^ ions. Such coexistence of emission lines originated from both optically active dopants is due to the energy transfer process from Tb^3+^ to Eu^3+^ [17,18,19,20,21,22]. In the case of the band recorded in a red spectral scope, a slight shift was observed of a maximum from 620 (XG_1Tb_) to 618 nm (XG_1Tb1Eu_), which was caused by overlapping the weak ^5^D_0_ → ^7^F_2_ band of Eu^3+^ ions (with a maximum at 615 nm) with the ^5^D_4_ → ^7^F_3_ band of Tb^3+^ ions (with a maximum at 620 nm). In general, the spectral matching of the donor’s emission (Tb^3+^) and the acceptor’s excitation (Eu^3+^) regions was a fundamental condition for energy transfer occurrence [62]. In this way, upon irradiation using the λ_exc_ = 351 nm line from NUV spectral region, Tb^3+^ ions could be successfully pumped into the ^5^L_9_ level, and then, the non-radiative de-activation to the ^5^D_4_ state took place. The excitation energy from the ^5^D_4_ state (Tb^3+^) could be successfully transferred into the ^5^D_1_ or the ^5^D_0_ level (Eu^3+^). Hence, among characteristic emission lines from Tb^3+^ ions, additional bands originated from Eu^3+^ can also be recorded.

**Figure 8:**
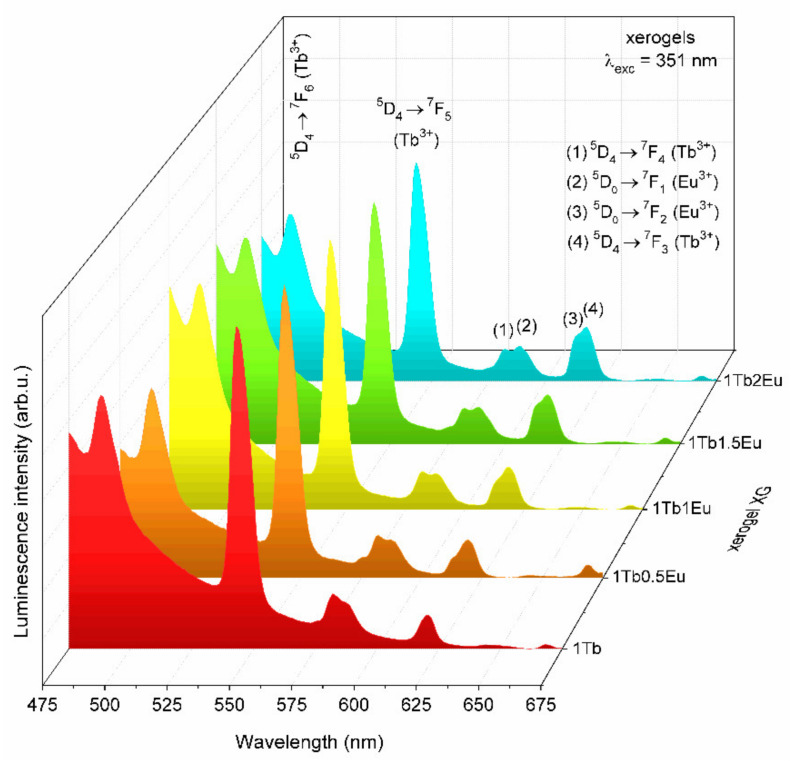
The emission spectra of XG_1Tb_ and XG_1TbxEu_ xerogels recorded under excitation at λ_exc_ = 351 nm.

The emission spectra of Tb^3+^/Eu^3+^ co-doped xerogels with the varying Tb^3+^:Eu^3+^ molar ratio recorded upon excitation at λ_exc_ = 351 nm are presented in Figure 8. A slight decrease was observed in the emission intensity of the ^5^D_4_ → ^7^F_6_ and the ^5^D_4_ → ^7^F_5_ bands of Tb^3+^ ions when the Tb^3+^:Eu^3+^ molar ratio gradually decreases. Hence, the R/G ratio values ((I(^5^D_0_ → ^7^F_2_)(Eu^3+^)+I(^5^D_4_ → ^7^F_3_)(Tb^3+^)/I(^5^D_4_ → ^7^F_5_)(Tb^3+^)) were estimated. For the XG_1Tb_ sample, the R/G ratio was defined as the ratio of integrated intensities of the ^5^D_4_ → ^7^F_3_ red band and the ^5^D_4_ → ^7^F_5_ green emission line [63]. In the case of the XG_1TbxEu_ co-doped samples, an additional contribution of luminescence originated from Eu^3+^ ions into total red emission should also be taken into account. Hence, the R/G ratio was calculated as (I(^5^D_4_ → ^7^F_3_)(Tb^3+^) +I(^5^D_0_ → ^7^F_2_)(Eu^3+^)/I(^5^D_4_ → ^7^F_5_)(Tb^3+^)) and its increase can be interpreted as a growing share of Eu^3+^ ions in total generated multicolor luminescence. Indeed, a slight increase in R/G ratio values was determined in the following order: From 0.09 (XG_1Tb_), through 0.15 (XG_1Tb0.5Eu_), 0.17 (XG_1Tb1Eu_), 0.26 (XG_1Tb1.5Eu_), to 0.30 (XG_1Tb2Eu_). Such an increment of the R/G ratio suggests more efficient Tb^3+^/Eu^3+^ ET when the Eu^3+^ content gradually grew, since the Tb^3+^:Eu^3+^ molar ratio changed from 1:0.5 to 1:2. Nevertheless, such a small increase in the R/G ratio resulted from the relatively large interionic distances between Tb^3+^ and Eu^3+^ ions, characteristic for the amorphous xerogel host.

Additionally, an increasing background for prepared sol-gel samples was also observed, especially at wavelengths <540 nm. Such a background was associated with the wide band from the silicate sol-gel host, as was shown by other authors, e.g., Tomina et al. [64], for different types of Eu^3+^-loaded aminosilica spherical particles. The authors suggest that such a band could result, i.e., from the charge transfer on Si-O bonds or defect from the silicate network. They have proven that such a wide band’s intensity is strictly related to the type of complexes formed by Eu^3+^ ions with amine ligands connected with the silicate sol-gel host. A similar effect was reported by Kłonkowski et al. [20], who synthesized sol-gel glass-ceramics containing SrF_2_ singly- and doubly-doped with Tb^3+^/Eu^3+^. The broad band’s origin was explained by defects, like dangling bonds, inside silicate sol-gel host.

**Figure 9:**
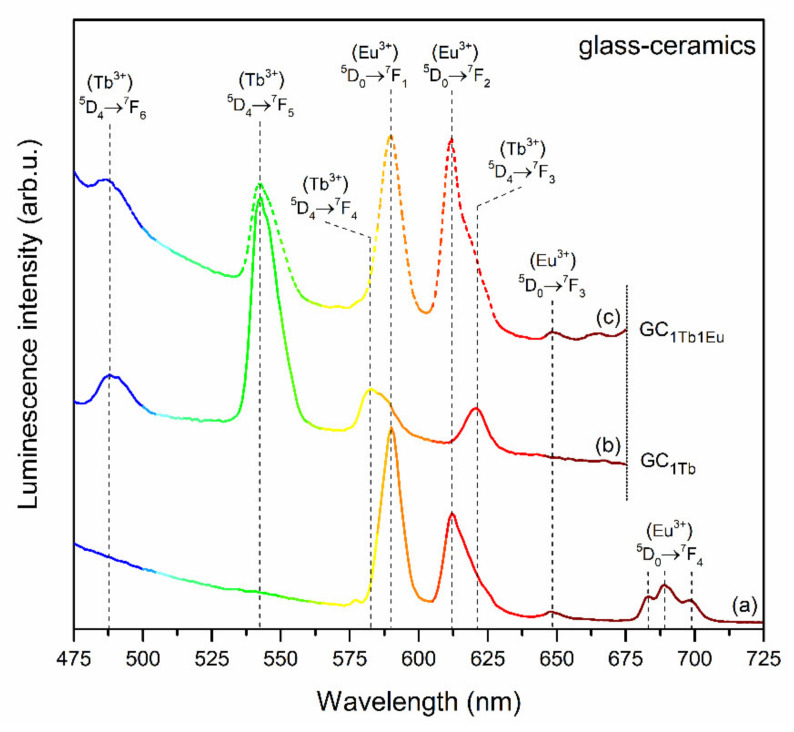
Emission spectra of: (**a**) GC_1Tb1Eu_ (λ_exc_ = 395 nm), (**b**) GC_1Tb_ (λ_exc_ = 351 nm), and (**c**) GC_1Tb1Eu_ (λ_exc_ = 351 nm).

The emission spectra recorded for GC_1Tb_ (upon excitation at λ_exc_ = 351 nm wavelength) and GC_1Tb1Eu_ co-doped the representative sample (recorded under excitation at λ_exc_ = 394 nm and λ_exc_ = 351 nm lines) are depicted in Figure 9. Similarly as for xerogel, for the GC_1Tb_ sample, the characteristic emission bands corresponding to the transitions from the ^5^D_4_ excited level into the ^7^F_6_ (488), ^7^F_5_ (542), ^7^F_4_ (581), and ^7^F_3_ (621 nm) lower-lying states were detected. In the case of the GC_1Tb1Eu_ sample, the coexistence of the luminescence lines originating from both rare-earth dopants was clearly observed after excitation at λ_exc_ = 351 nm line. Therefore, an appearance of characteristic emission bands coming from Eu^3+^ ions upon excitation of Tb^3+^ confirmed the occurrence of Tb^3+^/Eu^3+^ ET. It should be particularly pointed out that the intensity of Tb^3+^ emission strongly decreased, accompanied by significant enhancement of Eu^3+^ luminescence. Additionally, the maxima of bands recorded the in 570–630 nm spectral scope were shifted from 581 (for GC_1Tb_ sample) to 592 nm (for GC_1Tb1Eu_ sample) for an orange band and from 621 (for GC_1Tb_ sample) up to 612 nm (for GC_1Tb1Eu_ sample) for a red band. Indeed, an enhancement of Eu^3+^ emission via Tb^3+^/Eu^3+^ ET was much more effective for glass-ceramics than for xerogels.

**Figure 10:**
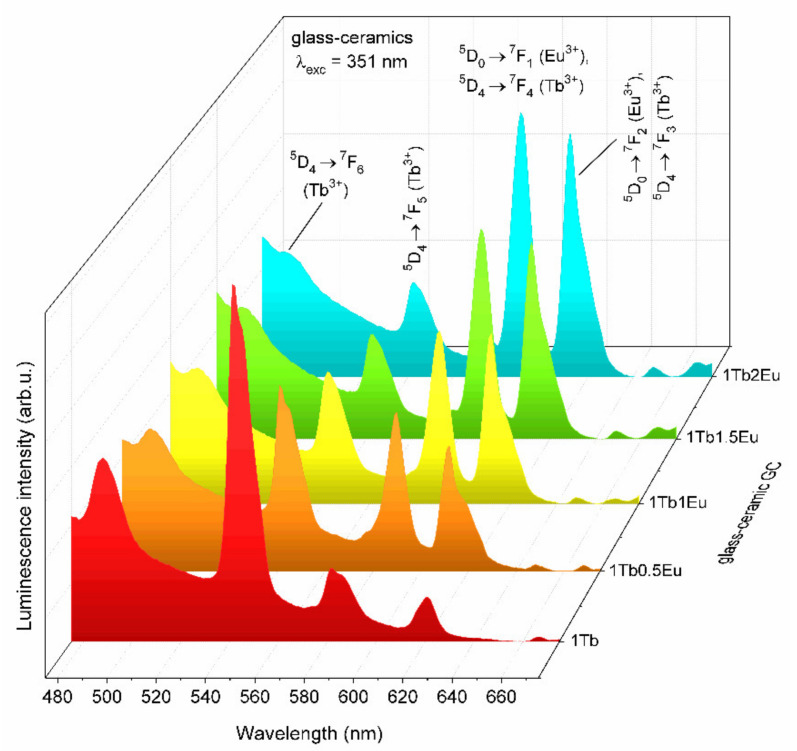
The emission spectra of GC_1Tb_ and GC_1TbxEu_ xerogels recorded under excitation at λ_exc_ = 351 nm.

The emission spectra recorded under λ_exc_ = 351 nm for GC_1TbxEu_ co-doped glass-ceramics are depicted in Figure 10. Based on the collected data, it was established that intensities of the ^5^D_4_ → ^7^F_J_ (J = 5,6) bands of Tb^3+^ in the bluish-green spectral scope are strongly dependent on the Tb^3+^:Eu^3+^ molar ratio. Indeed, the intensity of the Tb^3+^ emission was strongly reduced when the concentration of the acceptor gradually increased, and such an effect was simultaneously accompanied by a well-observable increase in the intensity of Eu^3+^ emission within the reddish-orange spectral scope. The observed correlations in mutual intensities of characteristic emission bands were accompanied by an adequate increase in R/G ratio values from 0.14 (GC_1Tb_) and 0.80 (GC_1Tb0.5Eu_), through 1.60 (GC_1Tb1Eu_), 2.47 (GC_1Tb1.5Eu_), and up to 3.76 (GC_1Tb2Eu_). Therefore, the increment in the calculated R/G ratio values was more dynamic for glass-ceramics than for xerogels, for which only a slight increase was reported when the Tb^3+^:Eu^3+^ molar ratio decreased. Such a correlation was undoubtedly associated with the decreased interionic distance between Tb^3+^ and Eu^3+^ ions due to their successful entering into the CaF_2_ crystal lattice.

For Tb^3+^ ions, the G/B ratio analysis defined as I(^5^D_4_ → ^7^F_5_)/I(^5^D_4_ → ^7^F_6_) could also be treated as a useful tool for characterization of the symmetry around Tb^3+^ dopant ions [65]. Since the ^5^D_4_ → ^7^F_5_ line is a magnetic-dipole in nature and the ^5^D_4_ → ^7^F_6_ transition is an electric-dipole one, the G/B ratio value should have changed when xerogels were transformed into glass-ceramic counterparts. Hence, the G/B ratio values should have been higher in more centrosymmetric sites [66]. Indeed, some changes in emission lines originated from Tb^3+^ ions could also be observed, similarly as for Eu^3+^. On the other hand, it should be pointed out that the G/B ratio was not as sensitive a spectroscopic probe as the R/O ratio calculated for Eu^3+^ optically active ions. The G/B ratio was calculated for samples singly-doped with Tb^3+^ ions, and the ratio changes from 2.95 (XG_1Tb_) to 3.80 (GC_1Tb_). The results were consistent with the data presented by us earlier in our previous work, concentrating on Tb^3+^-doped sol-gel materials’ photoluminescence behavior [67]. Based on structural changes undergone during controlled ceramization at 350 °C, the identified differences in G/B ratio values were clearly related to the migration of Tb^3+^ ions from the amorphous silicate sol-gel network into CaF_2_ nanocrystals formed during controlled heat-treatment.

#### 3.2.3. Effect of Changing in the Tb^3+^:Eu^3+^ Molar Ratio on Decay Times of the ^5^D_4_ (Tb^3+^)

The further evaluation of Tb^3+^/Eu^3+^ ET in fabricated sol-gel materials was based on the decay analysis of the ^5^D_4_ excited state of Tb^3+^ ions. Firstly, the interpretation of collected data allowed us to establish a clear correlation between the decay profile (mono- or double-exponential) and type of sol-gel material (i.e., xerogel or glass-ceramic). Indeed, the curves recorded for xerogels were well-fitted to a first exponential decay mode described by the following equation:

(3) $$I\left(t\right)\;={\;I}_{0}\exp\left(-t/\tau \right)$$

where I(t) and I_0_ are the luminescence intensities at time t and t = 0, respectively, while τ is the luminescence decay time [68]. Factually, in our xerogels, the rare-earths were chemically bonded with OH moieties and CF_3_COO^−^ anions in complex compounds [69]. It should be noted that high vibrational energies characterize such ligands, i.e., >3000 (OH groups) and ~1200, ~1140 cm^−1^ (CF_3_COO^−^ anions) as was demonstrated in the Structural characterization: XRD, TEM, and IR spectroscopy section (Figure 2). According to the energy gap law, the effective phonons with maximum energy located in a local surrounding of RE^3+^ ions (*ħω_max_*) generate the strongest effect on decay times [70]. In this case, since OH moieties’ vibrational energy was the highest, they played a major role in the non-radiative depopulation of excited states. For glass-ceramics, the decay curves were well-fitted to a second exponential decay mode, which can be expressed by the equation:

(4) $$I\left(t\right)/I_{0}=A_{1}\exp\left(-t/\tau_{1}\right)+A_{2}\exp\left(-t/\tau_{2}\right)$$

where A_1_ and A_2_ are amplitudes, while τ_1_ and τ_2_ are the decay times of short and long lifetime components, respectively [68]. The double-exponential decay profile, as well as considerable differences in τ_1_ and τ_2_ values, allowed us to conclude about the distribution of rare-earths between two chemically distinct surroundings characterized by different phonon energies. In fact, part of the RE^3+^ ions migrated during the controlled heat-treatment into the CaF_2_ crystal lattice, and formed inside the amorphous sol-gel network as a new chemical environment with low phonon energy (~466 cm^−1^). Due to such a low phonon energy of the CaF_2_ lattice, the multiphonon non-radiative depopulation of excited states was strongly restricted. However, the remainder of rare-earths was located in an amorphous sol-gel host. According to IR-ATR spectra recorded for glass-ceramics (Figure 2), it was observed that an intensity of the broad infrared signal originated from OH moieties was significantly reduced; therefore, a major role in non-radiative relaxation was attributed to Q^3^ groups (~1030 cm^−1^). Nevertheless, their phonon energy was greater than that of CaF_2_ crystal lattice. Such differences in phonon energies in the nearest surrounding of rare-earths determined the variable rates of radiative depopulation of their excited states: In silicate sol-gel host, the lifetimes were shorter (τ_1_ components), while in the CaF_2_ crystal lattice, the lifetimes were prolonged (τ_2_ components). Based on such distinguished lifetime components and their relative contributions to the total radiative decay profile, the average luminescence lifetime could be calculated using the following formula [71]:

(5) $$\tau_{\mathrm{avg}}=\;\frac{A_{1}\tau_{1}^{2}\;+{\;A}_{2}\tau_{2}^{2}}{A_{1}\tau_{1}\;+{\;A}_{2}\tau_{2}}$$

**Figure 11:**
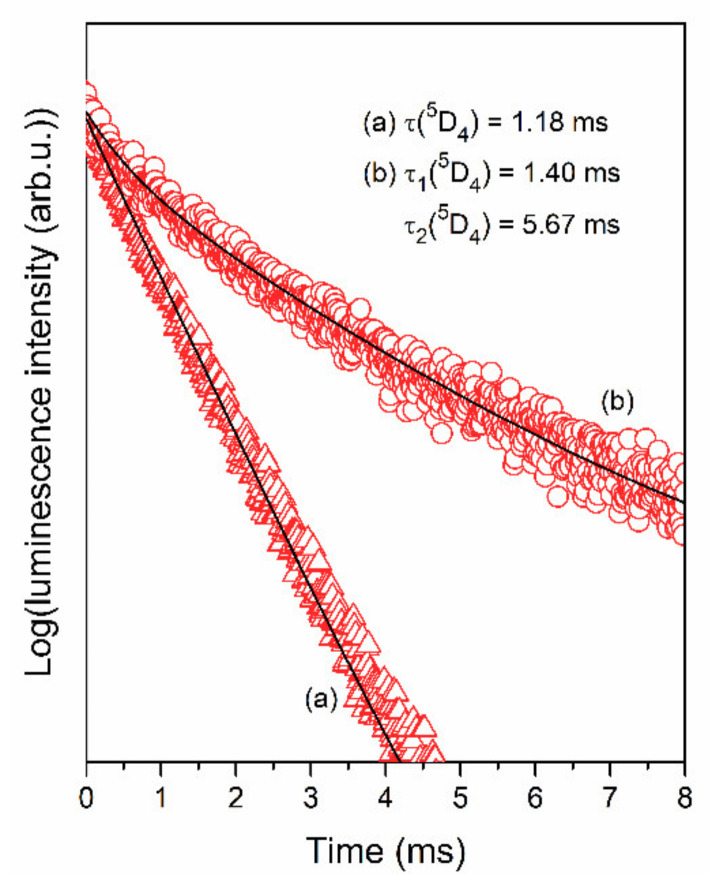
Luminescence decay curves of the ^5^D_4_ level of Tb^3+^ ions recorded for (**a**) XG_1Tb_, and (**b**) GC_1Tb_.

**Figure 12:**
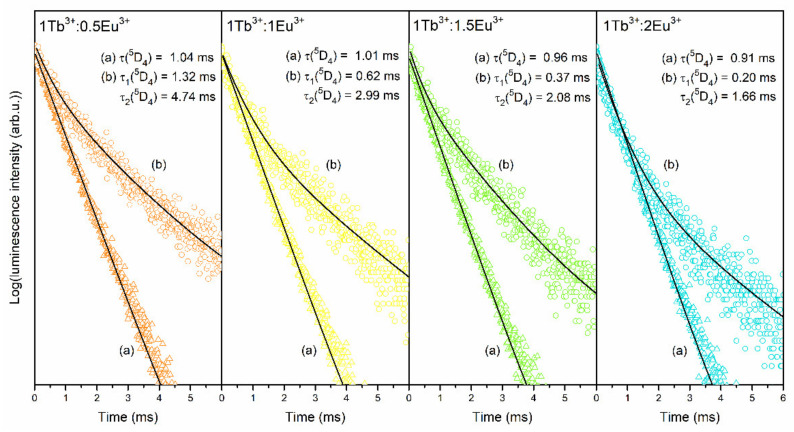
Luminescence decay curves of the ^5^D_4_ (Tb^3+^) level recorded for individual XG_1TbxEu_ and GC_1TbxEu_ co-doped samples: (**a**) Xerogels, (**b**) glass-ceramics. The curves were recorded upon excitation at λ_exc_ = 351 nm.

The luminescence decay curves of the ^5^D_4_ state (Tb^3+^) recorded for XG_1Tb_, GC_1Tb_, as well as for individual XG_1TbxEu_, GC_1TbxEu_ co-doped sol-gel samples are measured and plotted in Figure 11 and Figure 12, respectively. The decay curves were recorded upon λ_exc_ = 351 nm excitation and monitoring λ_em_ = 541 nm green luminescence of Tb^3+^ ions. A slight shortening of the decay lifetime was observed for xerogels from 1.18 (XG_1Tb_) to 1.04, 1.01, 0.96, and 0.91 ms for XG_1Tb0.5Eu_, XG_1Tb1Eu_, XG_1Tb1.5Eu_, and XG_1Tb2Eu_, respectively. For glass-ceramics, a change in the Tb^3+^:Eu^3+^ molar ratio from 1:0.5 to 1:2 resulted in significantly more efficient shortening of the average decay time of the ^5^D_4_ state from 4.75 (GC_1Tb_) to 3.75 (GC_1Tb0.5Eu_), 2.59 (GC_1Tb1Eu_), 1.92 (GC_1Tb1.5Eu_), and 1.55 ms (GC_1Tb2Eu_) (the individual values of τ_1_ and τ_2_ components are depicted in Table 1 and Table 2). Shorter luminescence lifetimes for xerogels than for glass-ceramics (when we compare the samples with the same Tb^3+^:Eu^3+^ molar ratio) were caused by the coordination of Tb^3+^ ions by high-vibrational OH groups, involved in the non-radiative depopulation of the ^5^D_4_ level. Their remarkable removal during controlled heat-treatment and partial segregation of Tb^3+^ inside CaF_2_ nanocrystals with low-phonon energy allowed the share of non-radiative processes in relaxation to be reduced significantly; hence, the lifetimes from the ^5^D_4_ state for glass-ceramics were longer.

Since the shortening in luminescence lifetimes was caused by an energy transfer from Tb^3+^ towards Eu^3+^ ions, the ratio of luminescence lifetimes of the ^5^D_4_ state of Tb^3+^ ions in the presence (τ) and the absence of Eu^3+^ ions (τ_0_) could be used as a valuable tool to estimate the energy transfer efficiency [72]:

(6) $$\eta_{\mathrm{ET}}=\;\left(1\;-\;\frac{\tau }{\tau_{0}}\right)\;\times \;100\%$$

For xerogels, the η_ET_ values increased from 11.86% (XG_1Tb0.5Eu_) through 14.41% (XG_1Tb1Eu_), 18.64% (XG_1Tb1.5Eu_) to 22.88% (XG_1Tb2Eu_). Compared to xerogels, a prompt increase in η_ET_ values has been noted for glass-ceramic materials, which reached 25.69% (GC_1Tb0.5Eu_), 45.47% (GC_1Tb1Eu_), 59.58% (GC_1Tb1.5Eu_), and 67.37% (GC_1Tb2Eu_). Hence, it was easily observed that the energy transfer efficiencies estimated for glass-ceramic samples were noticeably higher than for analogous xerogels with the same Tb^3+^:Eu^3+^ molar ratio, which was mainly caused by migration of rare-earths into the CaF_2_ crystal lattice during controlled heat-treatment, where the interionic Tb^3+^–Eu^3+^ distances were significantly shorter than in the amorphous sol-gel host. Additionally, those results clearly indicated a correlation between η_ET_ and a change in the Tb^3+^:Eu^3+^ molar ratio from 1:0.5 to 1:2 in fabricated sol-gel materials, which was undoubtedly associated with a higher probability that more Eu^3+^ ions could be located adjacent to Tb^3+^. Indeed, since the Tb^3+^/Eu^3+^ ET is characterized by dipole-dipole interactions [13,16], ET’s probability is proportional to 1/R^6^ (R is the average distance between Tb^3+^ and Eu^3+^). On this occasion, when Tb^3+^ and Eu^3+^ dopants were segregated inside CaF_2_ nanocrystal lattice, the interionic distances of Tb^3+^–Eu^3+^ pairs were vastly shortened notably if the Tb^3+^:Eu^3+^ molar ratio changed (from 1:0.5 to 1:2).

**Figure 13:**
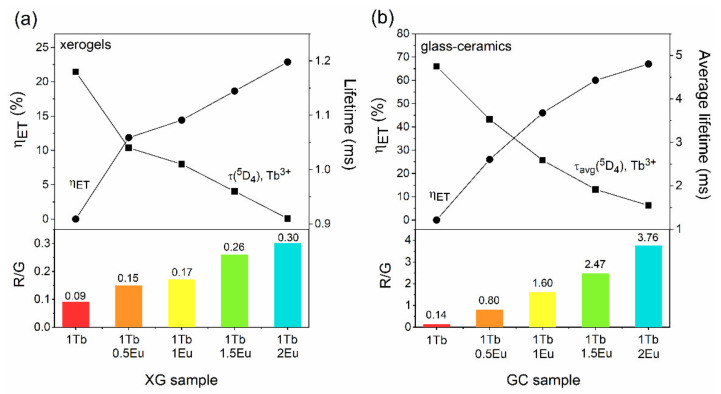
The relation between R/G ratio, energy transfer efficiency (η_ET_), and lifetime of the ^5^D_4_ (Tb^3+^) state for: xerogels (**a**) and glass-ceramics (**b**).

For better readability, the correlation between the R/G ratio, η_ET_, and τ(^5^D_4_) lifetimes for prepared sol-gel samples are graphically presented in Figure 13 and depicted in Table 1 (for xerogels) and Table 2 (for glass-ceramics). The R/G ratio gradually increased when the Tb^3+^:Eu^3+^ molar ratio changed from 1:0.5 to 1:2, pointing to the increasing share of emissions originated from Eu^3+^ ions, along with gradual growth in η_ET_ values and shortening of the τ(^5^D_4_) decay times of Tb^3+^ ions. For xerogels and glass-ceramic materials, such a relation was due to the increasing content of Eu^3+^ ions in accordance with Tb^3+^. The changes in the values of the parameters mentioned above for glass-ceramics materials were much more significant for each change of the Tb^3+^:Eu^3+^ molar ratio, which resulted from the preferential segregation of optically active ions into the CaF_2_ nanophase.

#### 3.2.4. The Luminescence Decay Analysis of the ^5^D_0_ State of Eu^3+^ Ions

**Figure 14:**
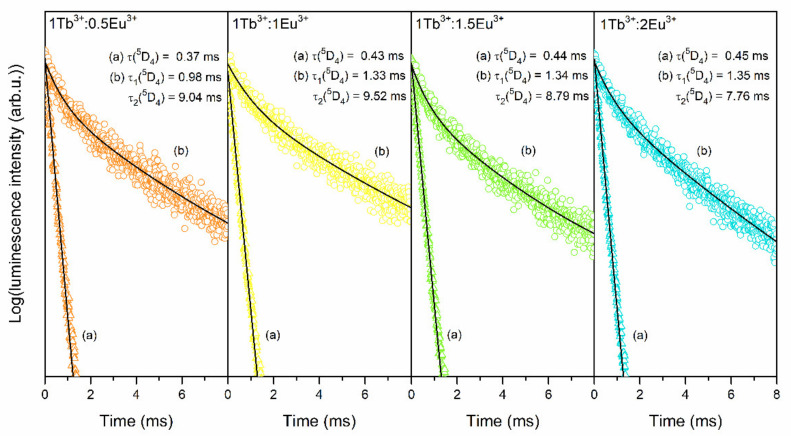
Luminescence decay curves of the ^5^D_0_ state of Eu^3+^ ions recorded for: (**a**) xerogels (λ_exc_ = 395) and (**b**) glass-ceramics (λ_exc_ = 394 nm).

The characterization of luminescence properties of fabricated Tb^3+^/Eu^3+^ co-doped sol-gel materials was supplemented by decay analysis of the ^5^D_0_ level (Eu^3+^) upon excitation at λ_exc_ = 394 wavelength and monitoring λ_em_ = 592 nm (Figure 14). The decay times were also depicted in Table 3 (for xerogels) and Table 4 (for glass-ceramics).

For xerogels, the τ(^5^D_0_) lifetime values hesitated from 0.37 (XG_1Tb0.5Eu_), 0.43 (XG_1Tb1Eu_), 0.44 (XG_1Tb1.5Eu_), to 0.45 ms (XG_1Tb2Eu_). The growing content of Eu^3+^ caused such a slight increase in decay times in prepared xerogels due to changing the Tb^3+^:Eu^3+^ molar ratio from 1:0.5 to 1:2. The relatively short luminescence lifetimes were caused by numerous OH groups in the immediate vicinity of Eu^3+^ ions in the silicate xerogel host. Interestingly, it was found that the τ_avg_(^5^D_0_) lifetimes in glass-ceramics exhibited no evident and straightforward correlation with the increasing content of Eu^3+^ ions as was found for xerogels. Indeed, the partial segregation of optically active dopants in CaF_2_ nanocrystals was responsible for the effective shortening of average distances between them and may have caused competition between radiative and non-radiative processes. Comparing the individual τ_avg_(^5^D_0_) lifetime values when the λ_exc_ = 394 nm wavelength was used as an excitation source, it was easy to observe that changing the Tb^3+^:Eu^3+^ molar ratio from 1:0.5 to 1:1 promoted the slight lifetime prolongation (τ_avg_(^5^D_0_) = 8.40 for GC_1Tb0.5Eu_ and τ_avg_(^5^D_0_) = 8.59 ms for GC_1Tb1Eu_). Meanwhile, a further change in the Tb^3+^:Eu^3+^ molar ratio (1:1.5 and 1:2) caused shortening of the calculated average decay time (τ_avg_(^5^D_0_) = 7.94 for GC_1Tb1.5Eu_ and τ_avg_(^5^D_0_) = 6.96 ms for GC_1Tb2Eu_). Since the R/O ratio values were almost the same for all fabricated glass-ceramic samples (from 0.59 to 0.64), we could assume that the relative distribution of Eu^3+^ ions between CaF_2_ nanocrystals and the amorphous sol-gel host was comparable in any case. Simultaneously, it also meant that the content of Eu^3+^ ions in precipitated CaF_2_ nanocrystals should have been proportional to the total concentration of Eu^3+^ introduced during the performed synthesis. Such a relation of the decrease in τ_avg_(^5^D_0_) values, when the Tb^3+^:Eu^3+^ molar ratio equaled 1:1.5 and 1:2, could be explained by the cross-relaxation process. In this case, an excited Eu^3+^ ion made a downward transition (^5^D_2_ → ^5^D_1_ and/or ^5^D_1_ → ^5^D_0_), whereas a coupled unexcited neighboring Eu^3+^ ion made an upward transition (^7^F_0_ → ^7^F_4_ and/or ^7^F_0_ → ^7^F_3_) [73]. Such non-radiative relaxation depended on the separation between Eu^3+^ interacting ions; hence, the shortening in the interionic distance inside CaF_2_ nanocrystals (promoted when the Tb^3+^:Eu^3+^ molar ratio exceeds 1:1) would be predominantly responsible for such a decrease in τ_avg_(^5^D_0_). To compare, in the case of Tb^3+^ ions, we excluded an involvement of the cross-relaxation process on luminescence lifetimes of the ^5^D_4_ state based on our previous results for SiO_2_-PbF_2_:Tb^3+^ sol-gel glass-ceramics, for which we reported the non-radiative relaxation mechanism when the molar ratio of Tb^3+^ (in accordance to Pb^2+^ cations) exceeded 0.6:1 [67]. On the other hand, since fabricated sol-gel samples were Eu^3+^ low-concentrated, such shortening of the τ_avg_(^5^D_0_) decay times when the Tb^3+^:Eu^3+^ molar ratio equaled 1:1.5 and 1:2 could be caused by lattice defects, which are well-known as quenching channels [74]. Indeed, a charge compensation induced the formation of vacancies inside the crystal lattice, the number of which would be greater if greater amounts of trivalent dopant ions entered into CaF_2_ nanocrystals [75]. Hence, the defects could be responsible for effective faster depopulation of the ^5^D_0_ state when the content of Eu^3+^ grows, resulting in shortening of the τ_avg_(^5^D_0_) decay times. Moreover, it is interesting to note that the τ_avg_(^5^D_0_) lifetime was prolonged when the Tb^3+^:Eu^3+^ molar ratio was achieved 1:1, and then reduced when the molar ratio equaled to 1:1.5 and 1:2, whereas the luminescence intensity was still increased. A similar effect was also observed for Eu^3+^-doped silicate hybrid materials [76]. The luminescence intensity successfully grew from 0.1 mol% up to 1 mol%; however, it was reported that the lifetimes of the ^5^D_0_ state gradually reduced from 617 (for 0.1 mol% Eu^3+^-doped sample) to 275 μs (for 1 mol% Eu^3+^-doped sample). In the case of our fabricated sol-gel samples, the experimental results from luminescence decay analysis have clearly proven that the variable molar ratio of Tb^3+^:Eu^3+^ and controlled crystallization of amorphous xerogels could be responsible for modulating the character of interionic processes.

Based on recorded emission spectra and performed decay analysis of the ^5^D_0_ state of Eu^3+^ ions, the quantum yields, Φ_Eu_, were calculated using Φ = k_R_/k equation. In this equation, k is the total decay rate constant (k = 1/τ(^5^D_0_)), whereas k_R_ is the radiative rate constant. The value of k_R_ was estimated from the following relation [77]:

(7) $$k_{R}=A_{\mathrm{MD},0}n^{3}\left(\frac{I_{\mathrm{tot}}}{I_{\mathrm{MD}}}\right)$$

where I_tot_ is the sum of integrated intensities of the ^5^D_0_ → ^7^F_J_ (J = 0–4) emission bands of Eu^3+^, I_MD_ is the integrated intensity of the ^5^D_0_ → ^7^F_1_ magnetic-dipole transition, n is the refractive index of the host and A_MD,0_ denotes the Einstein spontaneous emission coefficient for the ^5^D_0_ → ^7^F_1_ transition and its value for sol-gel systems is equal to 14.65 s^−1^ [78]. Similar to previous reports for CaF_2_ thin films [79] and CaF_2_ nanoparticles produced by the fluorolytic sol-gel process [80], the refractive index of CaF_2_ nanocrystals was close to n = 1.44. The quantum efficiencies for xerogels were very similar and hesitated from 9.3% to 10.5%. These values were changed drastically during controlled heat-treatment when xerogels were transformed into glass-ceramic systems. The quantum efficiencies achieved the following values: 75.6% (GC_1Tb0.5Eu_), 76.1% (GC_1Tb1Eu_), 69.0% (GC_1Tb1.5Eu_), and 60.0% (GC_1Tb2Eu_). Our calculations are in good agreement with results reported by Sun et al. [81] for Eu^3+^-doped CaF_2_ thin films, for which the highest quantum efficiency was estimated to 64.24%.

## 4. Conclusions

In summary, the Tb^3+^/Eu^3+^ ET was systematically investigated in a series of xerogels and glass-ceramics containing CaF_2_ nanocrystals and the variable Tb^3+^:Eu^3+^ molar ratio. The transformation of amorphous xerogels into glass-ceramics was successfully carried out at as low a temperature as 350 °C. A particular emphasis was placed on determining the correlation between the photoluminescence properties of prepared sol-gel materials and controlled crystallization, as well as the change in the Tb^3+^:Eu^3+^ molar ratio. The following points have been established:

- Using spectroscopy of Eu^3+^ ions as spectral probes, it was found that optically active dopants were preferably segregated inside the lattice of CaF_2_ nanocrystals during controlled heat-treatment of initial xerogels. Indeed, the ^5^D_0_ → ^7^F_1_ MD transition occupied the predominant advantage for glass-ceramics, which resulted in an almost six-fold decline in R/O ratio values from approximately 3.70 (for amorphous xerogels) to 0.64 (reported after controlled ceramization);
- The growing R/G ratio (from 0.09 to 0.30 for xerogels, and from 0.14 to 3.76 for glass-ceramics) was observed when the Tb^3+^:Eu^3+^ molar ratio changed from 1:0.5 to 1:2. Notably, in glass-ceramics, the emission of Tb^3+^ ions visibly gradually weakened, while luminescence of Eu^3+^ ions occupied the predominant advantage, significantly enhancing the reddish-orange emission;
- Performed decay analysis revealed an interesting dependence of decay times on change in the Tb^3+^:Eu^3+^ molar ratio, as well as partial segregation of Tb^3+^ and Eu^3+^ ions inside CaF_2_ nanocrystals formed during controlled heat-treatment at 350 °C. Indeed, a well-observable gradual shortening in τ(^5^D_4_) lifetimes for Tb^3+^ ions when the Tb^3+^:Eu^3+^ molar ratio changed from 1:0.5 to 1:2 was reported for xerogels (from 1.18 to 0.91 ms) and glass-ceramics (from 4.75 to 1.55 ms), and it was accompanied by an adequate increase in η_ET_ (from 11.9% to 22.9% for xerogels and from 25.7% to 67.4% for glass-ceramics). Higher η_ET_ values for the glass-ceramics resulted from a significant reduction in interionic distances between Tb^3+^ and Eu^3+^ ions inside the CaF_2_ crystal lattice;
- The decay analysis of the ^5^D_0_ state (Eu^3+^) clearly revealed that the partial crystallization induced a remarkable prolongation of τ_avg_(^5^D_0_) lifetimes even to 8.59 ms when the Tb^3+^:Eu^3+^ molar ratio equals 1:1, however, the further change in Tb^3+^:Eu^3+^ caused a slight shortening of decay times (7.94 when Tb^3+^:Eu^3+^ = 1:1.5, and 6.96 ms when Tb^3+^:Eu^3+^ = 1:2), which indicated a competition between radiative and non-radiative processes.

## Figures and Tables

**Table 1:** Measured lifetimes of the ^5^D_4_ state (Tb^3+^), energy transfer efficiencies, and R/G ratio values for prepared xerogels.

Xerogel	τ(^5^D_4_) (ms)	η_ET_ (%)	R/G
XG_1Tb_	1.18	-	0.09
XG_1Tb0.5Eu_	1.04	11.9	0.15
XG_1Tb1Eu_	1.01	14.4	0.17
XG_1Tb1.5Eu_	0.96	18.6	0.26
XG_1Tb2Eu_	0.91	22.9	0.30

**Table 2:** Measured lifetimes of the ^5^D_4_ state (Tb^3+^), average decay times, energy transfer efficiencies, and R/G ratio values for prepared glass-ceramics.

Glass-Ceramic	τ_m_(^5^D_4_) (ms)	τ_avg_(^5^D_4_) (ms)	η_ET_ (%)	R/G
GC_1Tb_	1.40 (τ_1_) 5.67 (τ_2_)	4.75	-	0.14
GC_1Tb0.5Eu_	1.32 (τ_1_) 4.74 (τ_2_)	3.75	25.7	0.80
GC_1Tb1Eu_	0.62 (τ_1_) 2.99 (τ_2_)	2.59	45.5	1.60
GC_1Tb1.5Eu_	0.37 (τ_1_) 2.08 (τ_2_)	1.92	59.6	2.47
GC_1Tb2Eu_	0.20 (τ_1_) 1.66 (τ_2_)	1.55	67.4	3.76

**Table 3:** Measured lifetimes of the ^5^D_0_ state (Eu^3+^) in xerogels (λ_exc_ = 395 nm excitation).

Xerogel	τ(^5^D_0_) (ms)
XG_1Tb0.5Eu_	0.37
XG_1Tb1Eu_	0.43
XG_1Tb1.5Eu_	0.44
XG_1Tb2Eu_	0.45

**Table 4:** Measured lifetimes and calculated average decay times of the ^5^D_0_ state (Eu^3+^) in fabricated glass-ceramics (λ_exc_ = 394 nm excitation).

Glass-Ceramic	λ_exc_ = 394 nm	
	τ_m_(^5^D_0_) (ms)	τ_avg_(^5^D_0_) (ms)
GC_1Tb0.5Eu_	0.98 (τ_1_) 9.04 (τ_2_)	8.40
GC_1Tb1Eu_	1.33 (τ_1_) 9.52 (τ_2_)	8.59
GC_1Tb1.5Eu_	1.34 (τ_1_) 8.79 (τ_2_)	7.94
GC_1Tb2Eu_	1.35 (τ_1_) 7.76 (τ_2_)	6.96

## Data Availability

The data presented in this study are available on request from the corresponding author.
